# 化妆品中禁用组分糖皮质激素样品前处理和分离分析研究进展

**DOI:** 10.3724/SP.J.1123.2025.01024

**Published:** 2025-12-08

**Authors:** Lian LIU, Xiaohua XIAO, Jihui FANG, Gongke LI

**Affiliations:** 1.中山大学化学学院，广东 广州 510006; 1. School of Chemistry，Sun Yat-sen University，Guangzhou 510006，China; 2.广东省药品检验所，国家药监局化妆品 风险评估重点实验室，广东 广州 510006; 2. Guangdong Institute for Drug Control，NMPA Key Laboratory for Safety Risk Assessment of Cosmetics，Guangzhou 510006，China

**Keywords:** 化妆品, 糖皮质激素, 样品前处理, 分析检测, cosmetic, glucocorticoid, sample preparation, analytical testing

## Abstract

糖皮质激素是机体应激反应重要的调节激素，也是临床上使用广泛而有效的抗炎和免疫抑制剂。化妆品中常非法添加糖皮质激素以增强其抗过敏、美白等功效，但化妆品基质复杂，糖皮质激素结构多样、含量低，其分析检测需要快速高效的样品前处理技术和灵敏准确的分析检测方法。本文综述了化妆品中糖皮质激素的样品前处理和分析检测方法的研究进展。其中样品前处理方法主要包括超声波辅助萃取、涡旋辅助萃取、电场辅助萃取等场辅助萃取法，液液萃取、液液微萃取等相分配方法，以及固相萃取、固相微萃取、分散固相萃取、磁固相萃取等相吸附方法。本文还重点介绍了化妆品中糖皮质激素的分析检测技术，主要包括色谱法、色谱-质谱联用法和毛细管电泳法以及包含光谱和显色法等在内的快速检测方法，展望了化妆品中糖皮质激素分离分析技术的发展趋势。

## Abstract (en)

Analyzing cosmetics is particularly important from a safety perspective owing to their widespread use in modern society. A wide range of ingredients and substances are restricted and/or prohibited to ensure that cosmetics are safe， with glucocorticoids among the prohibited substances. Glucocorticoids are important hormones that regulate the body’s stress response； they are also widely used as effective anti-inflammatory and immunosuppressive agents in the clinic. However， long-term topical use can lead to allergic reactions and other human-health effects； consequently， their inclusion in cosmetics formulations is prohibited. Glucocorticoids are often illegally abused in cosmetics with the aim of enhancing their anti-allergic and whitening effects. However， analyzing cosmetics is challenging owing to matrix complexity， structural diversity， and the trace-level （μg/kg） presence of glucocorticoids. Rapid and efficient sample pretreatment techniques and sensitive analytical methods are required to address these challenges， which necessitates separating/analyzing glucocorticoids and optimizing the separation/detection workflow. This review presents research progress into sample-pretreatment and analytical methods used to analyze and detect glucocorticoids in cosmetics during the 2010–2024 period. Sample preparation is a critical step when analyzing cosmetics because complex matrices can seriously interfere with the determination of target analytes； hence some separation or enrichment methods often need to be employed when analyzing glucocorticoids in cosmetics. These methods mainly include field-assisted extraction methods， such as ultrasonic-assisted， vortex-assisted， and electric-field-assisted extraction， phase-partitioning methods， such as liquid-liquid extraction and liquid-liquid microextraction， as well as phase-adsorption methods， such as solid-phase microextraction and solid-phase， dispersive-solid-phase， and magnetic-solid-phase extraction. Cosmetics are divided into three categories according to the matrix： aqueous， emulsion， and cream. Creams and emulsions contain many wax-based and lipid substances that often need to be separated and enriched by methods that involve binding field assistance or phase separation， with solid-phase extraction associated with the most （more than 40%） literature reports. Glucocorticoid dissolution is often accelerated by ultrasonic-assisted or vortex-assisted methods prior to solid-phase extraction or solid-phase microextraction. In addition， methods used to analyze and detect glucocorticoids in cosmetics mainly included liquid-chromatography， chromatography-mass spectrometry， and capillary electrophoresis in the 2010–2024 period， as well as rapid detection methods that are discussed in this review. Statistically， 64% of the reports in this timeframe use chromatography-mass spectrometry methods， with chromatography-related methods accounting for 85% of the methods used to analyze and detect glucocorticoids in cosmetics. Methods for rapidly testing glucocorticoids in cosmetics， including spectroscopy and colorimetry， among others， are also presented in this review owing to the sheer size of the market for beauty products and the high demand for on-site testing. With this background in mind， the separation and analysis of glucocorticoids in cosmetics are anticipated to show developmental trends. Currently， solid-phase extraction and other pretreatment technologies， along with liquid chromatography- and chromatography-mass spectrometry-based detection techniques are the main quality-control avenues for cosmetics. However， on the one hand， glucocorticoid structural diversity and the continuous discovery and use of new glucocorticoids necessitate the development of new analytical methods. In particular， integrating high-resolution mass spectrometry， nuclear magnetic resonance spectroscopy， and big-data-analysis methods when developing efficient qualitative and quantitative analytical methods is significantly important from a practical perspective. On the other hand， the development of rapid， efficient， and universal pretreatment technologies for glucocorticoids is key to effectively separating and analyzing them in cosmetics and is expected to receive increasing levels of attention. Furthermore， rapid analysis， especially analytical techniques suitable for rapid on-site screening， are expected to become focuses for the detection of glucocorticoids in cosmetics in response to the growing regulatory requirements of the cosmetics market. Integrating sample-pretreatment and analytical-detection methods， along with the development of visualized， integrated， and automated rapid-detection technologies， is expected to facilitate rapid on-site sampling and high-throughput cosmetics testing.

糖皮质激素（glucocorticoid）属于甾体类固醇激素，是环戊烷骈多氢菲基本母核^［1］^基础上经C1-C2位脱氢、C6-*α*位甲基化、C9-*α*位氟化、C11位羟基化等修饰后衍生而成的一系列化合物^［2］^，具有多种空间异构体。糖皮质激素类物质极性较强，易溶于水，lg *K*_ow_值通常在1.4~1.8，核心结构及常见类型如图1所示。

**图1:**
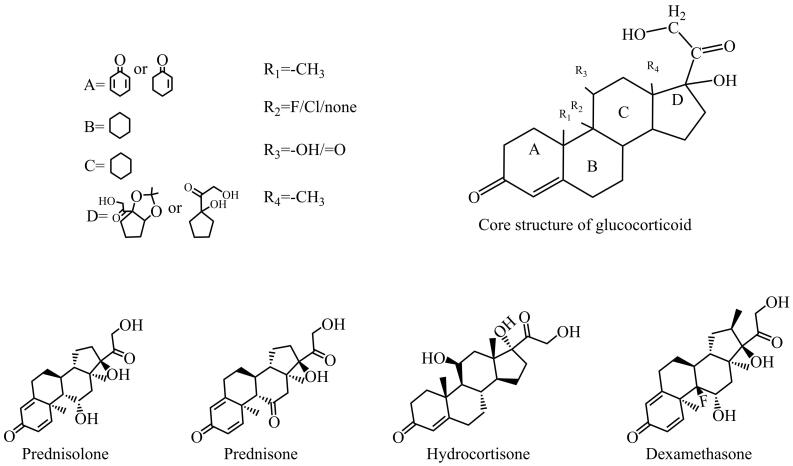
糖皮质激素核心结构及常见种类

糖皮质激素类物质具有抗炎^［3‒5］^、抗过敏^［6］^、免疫抑制^［7，8］^、抑制毛细血管扩张^［9，10］^等作用，在医药行业应用广泛。将糖皮质激素添加至化妆品中，可短期内降低毛细血管的通透性，产生“美白嫩肤”效果，两种及以上糖皮质激素组合添加能进一步实现短期内强效美白的效果^［11］^；糖皮质激素与抗感染药物（如甲硝唑、氯霉素等）联用、糖皮质激素与抗组胺药物（如氯苯那敏、苯海拉明等）联用^［12］^可以同时实现美白与抗感染、抗过敏效果；糖皮质激素与含汞化合物联用^［13］^可以放大美白效果等，但是长期使用含有糖皮质激素的化妆品，易引发依赖性皮炎^［14］^、皮肤萎缩^［15，16］^，大剂量使用甚至可能诱发高血糖^［17‒19］^、骨质疏松^［20‒23］^、库欣综合征^［24］^等，所以我国明确规定在化妆品中禁止添加糖皮质激素^［25］^。现行国家标准及相关管理文件如GB/T 24800.2-2009、GB/T 40145-2021、SN/T 2533-2010、药监局2016年第88号公告和2019年第66号公告、《化妆品安全技术规范》（2015版）中涵盖的化妆品中糖皮质激素主要有52种^［25-28］^。

化妆品种类多样^［29］^，从基底分类大致可归纳为水剂类、乳剂类、膏霜类、粉剂类等；按产品用途分类可分为洗护类、化妆类、美白类、祛痘类等。其中膏霜类与面膜类化妆品是糖皮质激素非法添加的重灾区^［13，30］^。近年来在实际抽查中屡有标准外糖皮质激素，甚至是新型糖皮质激素的检出^［31‒34］^，累计已超过100种。其中，最常检出的糖皮质激素包括泼尼松龙、泼尼松、氢化可的松和地塞米松等。

化妆品基质复杂，糖皮质激素结构多样、含量低，其分析检测需要快速高效的样品前处理技术和灵敏准确的分析方法以排除基体干扰并实现准确测定^［35］^。一般基质复杂的化妆品的样品制备可使用超声波辅助萃取（UAE）^［36］^等场辅助方法和液液萃取（LLE）^［37］^、固相萃取（SPE）^［38］^、固相微萃取（SPME）^［39］^等相分离手段，然后采取光谱法^［40］^、色谱法^［41，42］^等进行分析。有关化妆品分析的样品前处理技术进展，已有相关综述^［29，35，42］^报道，本文主要综述化妆品中糖皮质激素的样品前处理和分析检测方法的研究进展。

## 1 化妆品中糖皮质激素样品制备方法

化妆品中糖皮质激素的样品前处理方法主要包括超声波辅助萃取、涡旋辅助萃取（VAE）、电场辅助萃取（EAE）等场辅助萃取法，以及固相萃取、固相微萃取、分散固相萃取（DSPE）、磁固相萃取（MSPE）等相分离方法，图2列出了2010‒2024年化妆品中糖皮质激素的样品前处理相关文献的发表情况。

**图2:**
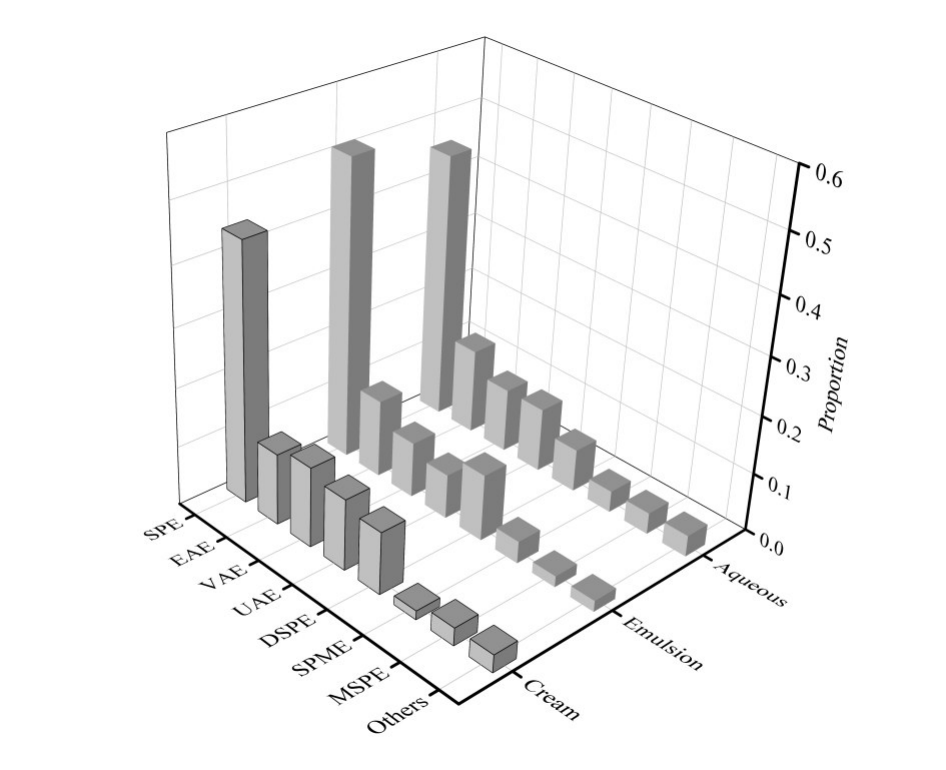
2010**-**2024年关于不同类型化妆品前处理方式发表的文献分布情况

化妆品种类繁多，样品基质复杂多变，糖皮质激素的添加量通常在μg/kg级别，且不同功效化妆品中使用的糖皮质激素结构和性能差异较大，因此，需根据化妆品的种类和基质选择合适的前处理方法。一般水剂类化妆品中蜡基、脂质类物质较少，前处理方法较为简单；膏霜、乳剂类化妆品中蜡基、脂质类物质较多，往往需要结合场辅助或者相分离等手段进行分离富集。

### 1.1 水剂类化妆品

水剂类化妆品包含水剂基质面膜类、精华水、化妆水、卸妆水等，此类化妆品中蜡基、脂质类物质和色素较少，一般以乙腈、甲醇等^［43，44］^为溶剂，利用涡旋使液液萃取更加充分，还可以结合超声波辅助萃取法实现糖皮质激素的有效分离富集。当样品中含有较高浓度的脂质成分时，采用乙腈、甲醇等萃取糖皮质激素时，脂类物质易溶于有机溶剂而对后续分析检测带来干扰。此时，采用离子液体（ILs）代替传统有机溶剂，发展均相液-液微萃取法^［45］^，利用亲水性ILs与离子对试剂在样品溶液中形成疏水性ILs，可实现化妆品糖皮质激素的高效分离富集，丙酸倍氯米松等3种糖皮质激素的检出限为0.54~1.66 ng/mL。李攻科等^［36］^发展的超声波辅助衍生一体化方法，仅需10 min即可快速分离和富集化妆品中氨基己酸等氨基酸；他们发展的膜保护微固相萃取-衍生一体化方法^［39］^，5 min即可完成化妆品中醛类化合物的富集和衍生化，这在化妆品痕量目标物分析中具有良好的应用前景，有望为糖皮质激素的快速分离富集提供新的思路。

电场辅助萃取法（EAE）利用电场作用提高微痕量分析物的萃取效率和选择性，但糖皮质激素在化妆品中一般呈电中性，难以直接使用EAE。借助带电微乳粒子、ILs等带电或可导电物质与糖皮质激素的相互作用或解离作用，可在电场下进行分离。如商少明等^［46］^利用带负电荷的十二烷基硫酸钠微乳粒子在电场作用下迁移的特性，通过电导率差异诱导糖皮质激素在溶液界面动态堆积，发展了一种大体积进样-非匀强电场扫集的在线富集微乳毛细管电动色谱方法，用于化妆品糖皮质激素的分析，使泼尼松、氢化可的松、泼尼松龙和倍他米松的富集倍数分别达853、893、933、925倍。陈新等^［47］^采用场放大进样方法将ILs作为添加剂引入微乳分离体系，利用ILs良好的热稳定性与溶解性从脂质基质中分离糖皮质激素，泼尼松、氢化可的松、醋酸可的松的富集倍数分别可达53、60、70；结合微乳液毛细管电动色谱技术在15 min内对这些糖皮质激素进行准确测定，且方法灵敏度可提高2个数量级。

在硼砂缓冲液中，调节溶液pH至碱性可使糖皮质激素解离^［48，49］^。基于此，商少明等^［50］^结合场放大堆积技术实现在线富集，泼尼松、氢化可的松、醋酸可的松、丙酸氟替卡松、曲安奈德、地塞米松的富集倍数分别为147、186、135、100、129和111，建立的毛细管电泳方法（CE）灵敏度比常规电泳方法提高了2个数量级。

水剂类化妆品的前处理技术近年来在糖皮质激素等痕量物质的分离富集方面取得显著进展，但仍面临着许多挑战。使用传统有机溶剂萃取虽操作简单、成本低，但对含高脂样品的选择性差，脂质干扰严重且环保性不足；而新型溶剂如ILs可通过调控亲疏水性显著提升选择性，但其合成成本高、稳定性受限，且需依赖离子对试剂增加操作复杂度。EAE借助带电微乳粒子或ILs介导，显著提高富集倍数，但对设备精度要求高，且高脂基质可能干扰电场稳定性。因此，针对脂质物质含量更高的乳剂和膏霜类化妆品，研究人员采取的方法与水剂类略有不同。

### 1.2 乳剂类化妆品

乳剂类化妆品包括洗发乳、身体乳、乳剂基质面膜等，由水相、油相、乳化剂组成，基质较水剂类化妆品更为复杂，其样品制备的要求也相应提高。SPE、SPME、DSPE或MSPE等相分离方法结合碳材料、印迹材料、多孔材料等分离介质的研究和应用较多。

涡旋、离心等力场辅助和声场辅助等简易场辅助手段在乳剂类化妆品处理中应用广泛。然而乳剂类化妆品中具有长烃链的化合物一般在配方中占比15%以上，超声-离心方法无法完全排除此类物质干扰，通常使用亚铁氰化钾-乙酸锌澄清剂沉淀大分子物质后结合SPE对杂质进一步去除。化妆品设计用途不同，其中添加剂不尽相同，致使化妆品基质理化性质存在差异，需对应选用合适的SPE柱以达到更好的去除效果，极性增强聚苯乙烯/二乙烯苯萃取柱（polar enhanced polymer）适于分离极性或中极性化合物，对一般基质的化妆品具有普适性^［51］^；Captiva EMR真菌毒素小柱（enhanced lipid removal）适于含长链烃类化合物的化妆品^［52］^；亲水亲脂平衡柱（hydrophile-lipophile balance）适于极性范围大的化妆品^［53，54］^；十八烷基硅烷键合硅胶填料柱（octadecylsilyl）适于含脂类、生物大分子较多的化妆品^［55］^。

与常规SPE相比，整体柱具有更好的多孔性和通透性。贾琼团队^［56］^采用石墨烯纳米片功能化修饰制备了聚（甲基丙烯酸丁酯-二甲基丙烯酸乙二醇酯）整体柱，用于分离富集化妆品中地塞米松等9种糖皮质激素。结果表明，石墨烯修饰的整体柱对9种糖皮质激素的富集能力相较于未修饰的甲基丙烯酸丁酯柱有显著提升，且在重复利用30次后萃取效果未明显下降。Wei等^［57］^结合原位光聚合反应法制备了多孔整体聚合物微萃取棒，如图3所示。该微萃取棒由乙二醇二甲基丙烯酸酯、2，2-偶氮二异丁腈和甲基丙烯酸经“一步一锅法”合成，方法简便快捷，对泼尼松、泼尼松龙、醋酸泼尼松龙3种糖皮质激素有较好分离效果，整体性能与商品化亲水亲脂平衡柱相当。

**图3:**
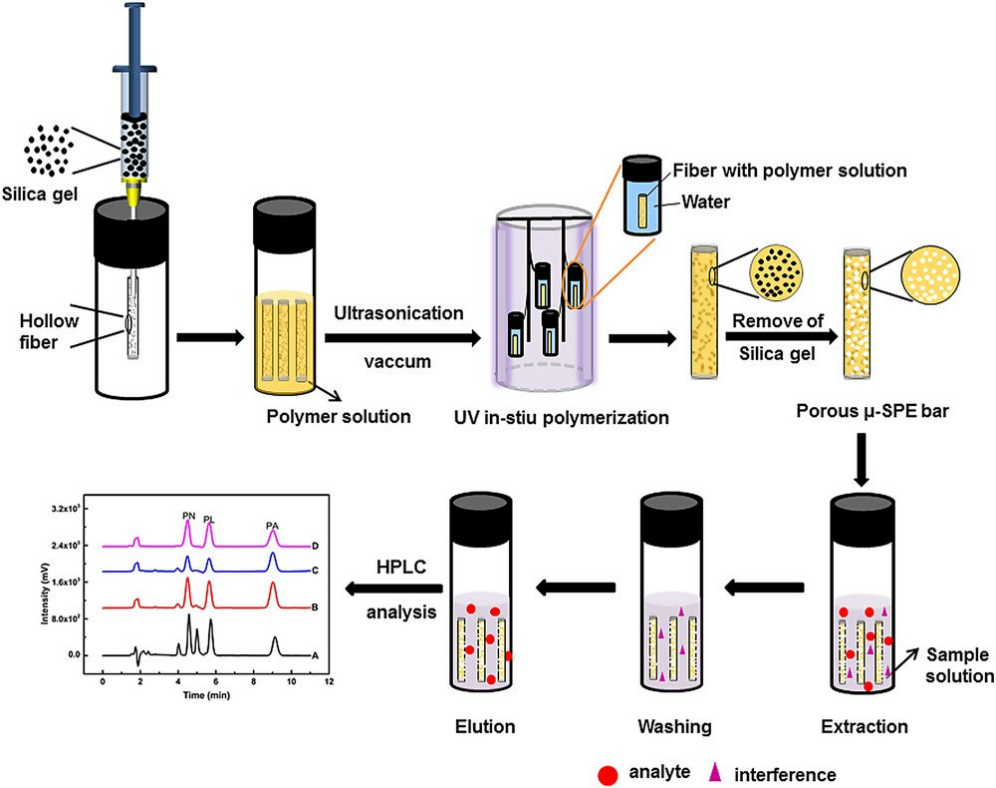
多孔整体聚合物萃取棒的制备和微固相萃取流程示意图^［57］^

相较于SPE，DSPE操作简便，萃取时间更短。为提高DSPE的选择性，Fu等^［58］^结合分子印迹技术（MIPs），以地塞米松为模板分子合成了单分散分子印迹微球，特异性地分离化妆品中痕量地塞米松，印迹因子为2.04。QuEChERS是经典的DSPE，常用吸附剂有C_18_^［1，59，60］^、*N*-丙基乙二胺^［1，59，60］^、石墨化炭黑（GCB）^［59］^等，可以排除化妆品中增溶剂、色素等杂质对糖皮质激素富集效果的影响。一般认为GCB对于平面分子的良好吸附性会使其在吸附杂质的同时对糖皮质激素造成损失，而阳胜等^［59］^发现，由于存在竞争吸附，少量GCB的加入反而可减少基质中苯环等平面构型分子产生的干扰。

MSPE通过外加磁场无需过滤离心即可实现吸附剂与样品的分离，简便快捷。Fu等^［61］^在磁性Fe_3_O_4_@SiO_2_纳米粒子表面以地塞米松为模板分子制备了磁性MIPs，可选择性地快速萃取护肤品中的地塞米松，印迹因子为2.05。Liu等^［62］^使用氢化可的松和地塞米松作为双模板分子，通过表面聚合法在Fe_3_O_4_@SiO_2_-NH_2_表面合成了双模板磁性MIPs，可同时有效分离富集化妆品内低浓度的氢化可的松和地塞米松，印迹因子分别为3.15和3.46。李攻科等^［63］^制备了一种类石墨碳氮化物材料（Fe_3_O_4_/g-C_3_N_4_/MIL-101）功能化的磁性MOF-101，如图4所示，比表面积和孔体积分别从Fe_3_O_4_/C_3_N_4_的190 m^2^/g 和0.052 cm^3^/g 提高到710 m^2^/g 和0.14 cm^3^/g，利用g-C_3_N_4_与糖皮质激素间的氢键作用和MIL-101的尺寸匹配效应实现5种糖皮质激素的高效富集。他们据此发展的MSPE-UPLC方法，灵敏度与其他检测化妆品中糖皮质激素的同类方法^［64］^相比提高了2个数量级以上。

**图4:**
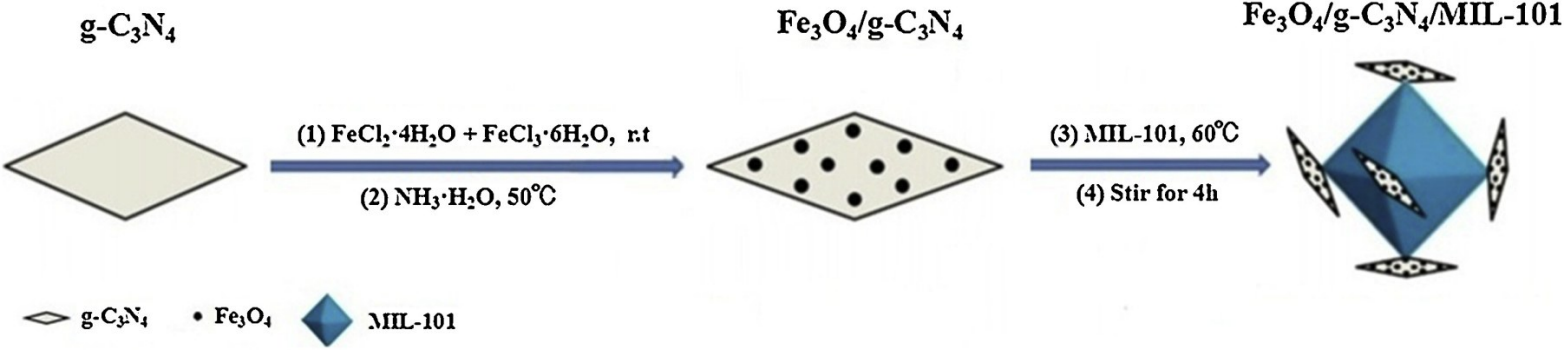
Fe_3_O_4_/g-C_3_N_4_/MIL-101合成示意图^［63］^

乳剂类化妆品因基质复杂，其前处理需结合针对性分离技术以应对高含量长烃链化合物及多样化添加剂干扰。目前常用方法中，SPE通过特定功能性填料可选择性去除脂类或极性杂质，但常规SPE步骤烦琐，耗时较长；DSPE操作简便、耗时短，但传统吸附剂如GCB可能导致目标物损失，需优化吸附剂用量以平衡杂质去除效果与回收率。MIPs可显著提升选择性，但制备工艺复杂且成本较高。MSPE凭借Fe_3_O_4_基材料实现快速磁分离，功能化修饰的同时利用氢键、尺寸匹配等效应，增大比表面积并提高富集灵敏度，但磁性复合材料合成技术要求高，且可能引入新干扰。整体柱技术具有多孔性和通透性优势，但规模化制备难度大。未来需进一步开发低成本、高稳定性吸附材料如多功能磁性MOFs等，并向前处理自动化发展。

### 1.3 膏霜类化妆品

膏霜类化妆品通常指发膏、膏霜、口红、睫毛膏等含高级脂肪醇/酸、羊毛脂系列衍生物、硅油系列衍生物等脂质类物质和蜡基较多的化妆品。膏霜类化妆品较水剂、乳剂类的脂质含量更多，更接近固态，在液液萃取前需先使用饱和氯化钠进行分散，再依靠超声、涡旋等场辅助手段加速糖皮质激素的溶出，其中的大分子杂质可以加入澄清剂沉淀除去或通过多次萃取-离心取上清液的方式简单除去。此外，仅使用LLE时，膏霜类样品中的糖皮质激素类物质的回收率较低^［51］^，通常还需辅以SPE、DSPE^［60，65］^等分离富集技术以去除如色素等杂质。

SPE是较成熟的净化各类基质化妆品的方法，研究表明，在SPE步骤前引入冷冻离心步骤或采用亚铁氰化钾-醋酸锌溶液沉淀乳化基质^［66］^，可排除提取液中的脂类干扰物，或采用过滤型固相萃取柱^［67］^、无需活化与平衡即可有效排除脂溶性物质的干扰。刘红等^［68］^使用高通量全自动固相萃取仪对化妆品样品进行净化，实现了无人值守下的固相萃取，结合超高效液相色谱-串联质谱法（UPLC-MS/MS）完成了76种糖皮质激素的测定，提高了方法准确性。为提升SPE的选择性，Li等^［69］^以泼尼松为模板分子，结合涂覆有多壁碳纳米管的聚乙烯过滤板用作SPE吸附剂，如图5所示。该吸附剂对泼尼松的吸附容量较相应的非分子印迹聚合物差值可达10.65 mg/g，显著大于对地塞米松等3种结构类似物的吸附容量差值（0.73~2.54 mg/g），可实现膏霜类化妆品中泼尼松的选择性吸附分离。

**图5:**
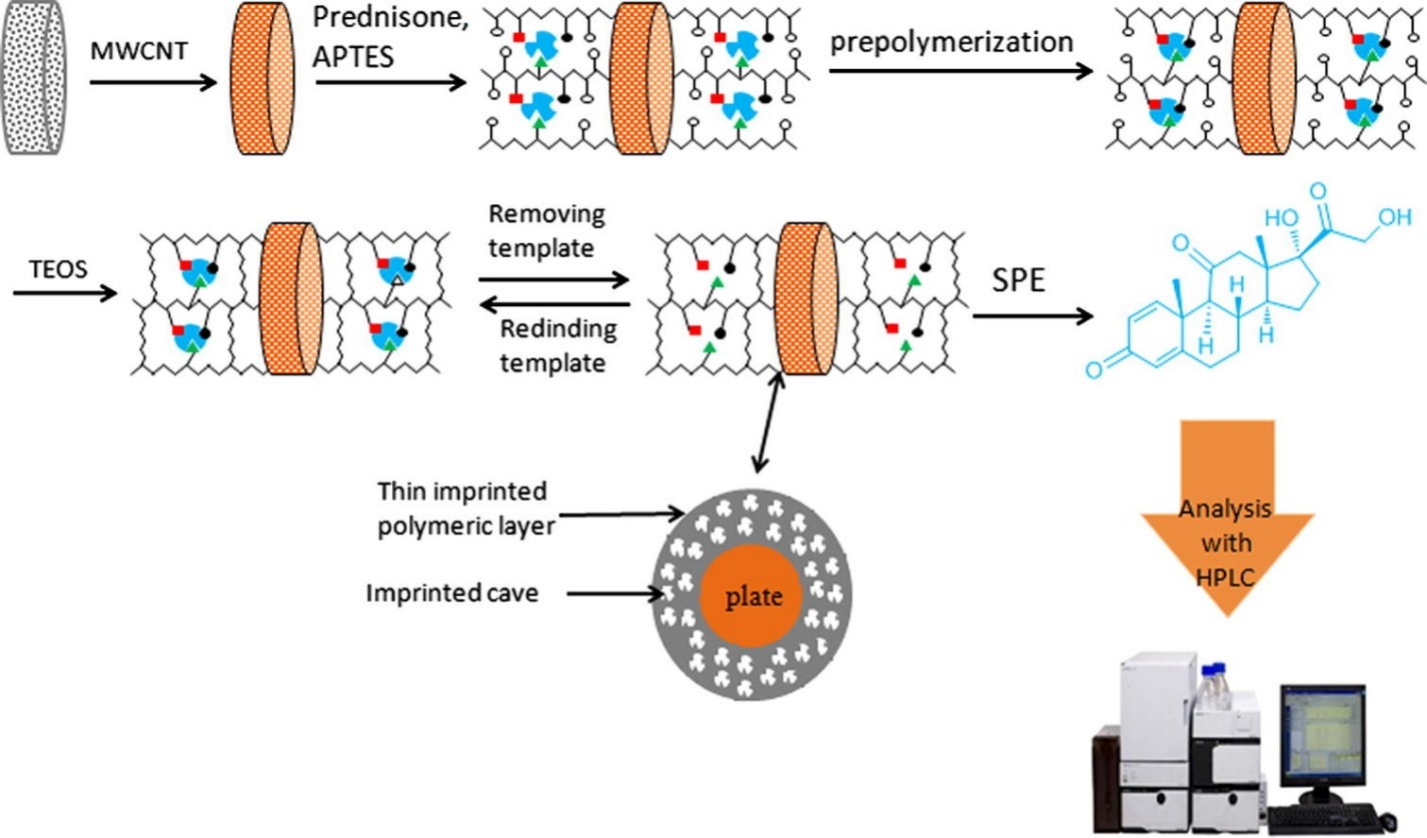
泼尼松分子捕获单体的合成及应用程序^［69］^

膏霜类化妆品在3类化妆品中基质最为复杂，基质效应对方法灵敏度、精密度、准确度均有影响，罗辉泰等^［65］^在使用DSPE并优化净化条件后发现，膏霜类化妆品中绝大多数糖皮质激素基质效应的绝对值在20%以内，随后，他结合同位素内标法实现了81种糖皮质激素的测定。

除了SPE等前处理方法外，崔晗等^［70］^报道了一种基于环己烷-乙酸乙酯体系的凝胶渗透色谱方法，用于化妆品中激素类禁用组分的分离富集，并结合LC-MS/MS对曲安西龙等15种激素进行了同时测定。

膏霜类化妆品因高脂质、蜡基含量及近固态基质特性，前处理环节需克服糖皮质激素回收率低、脂质干扰严重等难题。传统液液萃取法依赖超声/涡旋辅助以提升溶出效率，通常还需结合SPE或DSPE技术增强净化效果。SPE中引入冷冻离心或亚铁氰化钾-醋酸锌沉淀可有效去除脂类干扰，过滤型SPE柱及全自动高通量仪器可显著提升处理效率与准确性，但常规SPE柱对复杂基质如高蜡含量的普适性仍受限；MIPs选择性优于非印迹材料，但对于结构类似物的分离仍需优化。DSPE通过优化吸附剂组合可控制基质效应，但吸附剂选择与用量需谨慎，以避免竞争吸附导致的损失。

未来需继续开发高选择性吸附材料、集成自动化前处理平台，并针对固态基质特性优化分散-萃取协同工艺，以兼顾效率、灵敏度与复杂脂蜡体系的适应性；聚焦多技术联用、绿色溶剂开发，建立针对不同化妆品体系的通用化方案，以突破成本、环保性与复杂基质适应性的瓶颈，为化妆品痕量有害物质的高效筛查提供更优解决方案。

## 2 化妆品中糖皮质激素的分析方法

化妆品中糖皮质激素的分析检测，国家规定的标准方法主要有薄层色谱法、LC、LC-MS和毛细管电泳等^［26，27］^。液相色谱-质谱联用的方法常用于对样品进行定性定量或对新方法进行仲裁分析。对2010-2024年间化妆品中糖皮质激素的分析检测相关文献的发表情况进行调研可知，与色谱相关的文献占85%，其中色谱-质谱联用法的使用情况达64%。除此之外，由于化妆品市场日益扩大，现场检测需求增大，化妆品中糖皮质激素等禁限用组分的快速检测方法受到越来越多的关注。

### 2.1 色谱法

#### 2.1.1 液相色谱法

色谱法因其良好的分离度、极低的检出限被广泛应用于分析痕量有机物。液相色谱法是化妆品中糖皮质激素的经典分离定量方法^［28］^，一般配备二极管阵列检测器或双波长紫外检测器，用外标法定量。在液相色谱法的基础上，为提高柱效与化妆品样品分析速度，引入了高效液相色谱法^［54］^与反相高效液相色谱法^［71］^。表1中列出了色谱法在化妆品中糖皮质激素分析中的应用。

**表1:** 色谱法在化妆品中糖皮质激素分析中的应用

No.	Method	Number of analyte species	LOQ/（μg/kg）	LOD/（μg/kg）	Linear range/（μg/L）	Ref.
1	LC	6	/	0.78‒1.12^*^	1000‒200000	［^54^］
2	LC	9	2.00^*^	1.00^*^	50‒10000	［^71^］
3	LC-MS	3	100	/	1.0‒100	［^44^］
4	LC-MS	3	/	2.00‒4.00	50‒800	［^72^］
5	LC-MS	4	6	2	2.0‒50	［^73^］
6	LC-MS	13	3.3‒9.9	1.00‒3.00	1.0‒20	［^74^］
7	LC-MS	16	10	2.20‒3.30	1.0‒100	［^60^］
8	LC-MS	41	3.0‒7.0	1.00‒2.00	1.0‒200	［^75^］
9	LC-MS	42	2.0‒10	/	1.0‒200	［^76^］
10	LC-MS	47	/	50.0‒400	/	［^77^］
11	LC-MS	48	100	30	2.5‒60	［^78^］
12	LC-MS	50	100‒300	0.03‒0.10^*^	6.0‒60	［^79^］
13	LC-MS	83	2.0‒76	1.00‒23.0	2.0‒200	［^80^］
14	LC-MS	100	100	30	2.5‒60	［^81^］
15	LC-MS	8	170‒1120	50‒340	200‒10000	［^82^］
16	LC-MS	12	3.0‒5.0	1.0‒2.0	2.0‒500	［^64^］
17	LC-MS	41	10	/	1.0‒100	［^83^］
18	LC-MS	1	0.03^*^	0.01^*^	0.5‒500	［^84^］
19	CE	4	/	12.0‒20.0^*^	40‒16000	［^85^］
20	CE	3	/	900‒1200^*^	5000‒400000	［^86^］
21	CE	6	/	660‒860^*^	2000‒500000	［^87^］
22	CE	3	/	1000‒1300^*^	5000‒100000	［^88^］

* μg/L； /： no data.

色谱法虽然可以对糖皮质激素进行分离定量，但仅能鉴别已知目标物，目前尚无单独使用液相色谱方法对未知糖皮质激素定性的标准方法，而糖皮质激素种类繁多，现有文献和标准多基于已知物质，可能导致新型糖皮质激素未被检出。液相色谱-质谱联用方法结合了液相色谱的分离定量能力与质谱的定性能力，成为化妆品中糖皮质激素检测的通用方法。

赵晓亚等^［71］^使用反相高效液相色谱法对化妆品中9种糖皮质激素进行检测，线性范围为0.05~10.00 mg/L，检出限为1.0 μg/kg，回收率达85.0%~99.5%，相对标准偏差小于5.8%，具有高灵敏度、可重复性和高效分离能力，尤其适用于复杂基质中痕量已知糖皮质激素的定量分析；但其依赖标准品建立外标曲线，仅能针对预设目标物进行定性定量，无法识别新型或结构未知的糖皮质激素。这一局限性凸显了单一色谱法的不足，需结合质谱联用技术以拓展未知物的筛查能力，从而更全面地保障化妆品安全监管的精准性。

#### 2.1.2 色谱-质谱联用法

质谱方法具有选择性好、灵敏度高、抗干扰强的特点^［75］^，与色谱结合不仅可以实现目标物的高灵敏度定量分析，也可以利用质谱实现目标物的结构鉴定。国家标准方法中规定了使用液相色谱^［27］^/高效液相色谱-串联质谱法^［26］^测定，外标法定量化妆品中糖皮质激素的方法。在实际检测过程中研究人员不断对前处理^［60，75，76］^、色谱柱^［72，73，78，80，89］^、流动相^［74］^、质谱^［77，79］^等条件进行优化，目前已经对至少100种糖皮质激素建立了定性定量方法。

李薇等^［90］^建立了一种以四极杆飞行时间质谱技术为基础的检测方法，经过一级、二级质谱解析，结合碱水解后产物保留时间，可用于倍他米松衍生酯的定性，并对倍他米松丁酸丙酸酯进行了定量测定。将高分辨质谱与糖皮质激素数据库及谱库检索功能结合，只需一次进样即可实现对化妆品中糖皮质激素的高通量快速筛查，无需标准样品^［1］^。与串联质谱相比，高分辨质谱具有更强的定性能力与更高的灵敏度，且能有效区分质荷比相近的干扰物，减少假阳性结果的出现^［75，81］^。目前应用于化妆品中糖皮质激素检测的高分辨质谱主要包括飞行时间质谱^［82］^、静电轨道阱组合式质谱^［64，83］^等。此外，单独使用液相色谱-三重四极杆质谱法、液相色谱-四极杆飞行时间质谱法或结合核磁共振与单晶X射线衍射技术^［31］^等也可对未纳入标准或新型糖皮质激素进行定性，如氯倍他索乙酸酯^［31-33］^、倍他米松丁酸乙酸酯^［34］^等。我们基于自建禁限用组分高分辨质谱库对化妆品进行筛查检验，实现非法添加禁用物质的快速筛查，成功在两批祛斑类化妆品中筛查到标准外糖皮质激素甲基泼尼松^［43］^。

此外，超高效合相色谱同样可用于糖皮质激素同分异构体，尤其差向异构体的拆分，且分离效果良好。阳胜等^［59］^建立了以超临界CO_2_为主要流动相的超高效合相色谱法-串联质谱法，分析了包含曲安西龙双醋酸酯在内的45种糖皮质激素，线性范围为0.20~100 μg/L，LOD为0.05~0.10 μg/kg。

色谱-质谱联用技术在化妆品糖皮质激素检测中展现出显著优势：通过结合液相色谱的高效分离与质谱的高灵敏度、高选择性，不仅可实现痕量目标物的精准定量，还能借助多级质谱解析碎片离子，完成结构鉴定与未知物定性。此外，三重四极杆质谱可通过多反应监测模式提升抗干扰能力，而静电轨道阱质谱可凭借超高分辨率区分质荷比相近的干扰物。然而，色谱-质谱联用技术存在设备成本高昂、操作复杂、依赖专业技术人员等问题，且部分方法耗时较长。

#### 2.1.3 毛细管电泳法

毛细管电泳法分离效果好，分析速度快，是化妆品中糖皮质激素分析的主要技术之一。微乳毛细管电动色谱法对中性疏水物质分离效果较好，在进行化妆品中糖皮质激素分析时常辅以如*β*-环糊精^［85］^、ILs^［86，87］^、鼠李糖脂^［88］^等添加剂以改善分离选择性或稳定微乳体系。Cao等^［91］^使用具有核壳结构的两亲性无规共聚物聚（甲基丙烯酸甲酯-甲基丙烯酸）作为毛细管电泳的新型假固定相，该假固定相是脂质基质中糖皮质激素的良好载体，提高了选择性和稳定性，方法用于8种糖皮质激素的分析，LOD为0.33~0.76 mg/L。

毛细管电泳法凭借其高分离效率、快速分析及低试剂消耗的优势，在化妆品糖皮质激素检测中展现出独特潜力，尤其是微乳毛细管电动色谱通过引入*β*-环糊精、ILs等添加剂，有效提升了中性疏水目标物糖皮质激素的分离选择性和体系稳定性。然而，毛细管电泳法的灵敏度通常低于色谱-质谱联用技术，且对高脂/蜡基等复杂基质的适应性较弱，需依赖样品前处理优化或功能化添加剂以增强抗干扰能力；相较于色谱法的高灵敏度及色谱-质谱联用技术的未知物定性能力，毛细管电泳法虽在结构解析方面存在局限，但适于快速筛查已知目标物。而与此同时，为推动检测场景多元化，全面监管化妆品中糖皮质激素，更多快检方法正逐渐兴起。

### 2.2 快速检测法

美妆市场产能激增，样品量庞大，质量参差不齐，而实验室的定性定量分析容量有限，因此化妆品的快速分析方法需求急剧上升。快速分析一般可分为实验室快速分析方法和现场快速检测方法，含前处理在内前者通常在2 h内完成一次分析，后者通常要求在0.5 h内完成。为提升分析速度，实现现场快速筛查，在经典液相色谱-质谱联用方法之外，化妆品中糖皮质激素的快速检测方法如光谱法、电化学法、显色法与酶联免疫吸附法等，得到越来越多的应用。

现行标准中规定的薄层色谱法^［26］^采用茴香醛显色法和氯化四唑蓝显色法技术，可定性41种糖皮质激素；陈喆等^［92］^选用氯化硝基四氮唑蓝为显色剂，可快速筛查曲安西龙等28种糖皮质激素，LOD为1 μg/mL，可保证较高检测正确率，适于现场大样本量初步筛查。

膏霜类化妆品因自身分子流动性受限，应用传统光谱方法进行分析往往谱线宽且分辨率低，而高分辨率-魔角旋转核磁共振光谱技术可直接得到这类样品的质子光谱，Vermathen等^［93］^借此成功实现无萃取或色谱分离步骤直接检测膏霜类化妆品中醋酸地塞米松，灵敏度可达0.01%。

糖皮质激素氧化/还原性较弱、电化学活性较差，以电化学方法对其进行筛查的报道非常少。Luo等^［94］^通过两步原位电聚合反应制备了聚-L-丝氨酸/聚-牛磺酸改性电极，制备过程如图6所示。以聚牛磺酸为选择性检测倍他米松的识别单元、聚-L-丝氨酸对倍他米松进行改性，提高了电化学活性，可用于直接测定化妆品中的倍他米松，检出限为32.2 ng/mL。

**图6:**
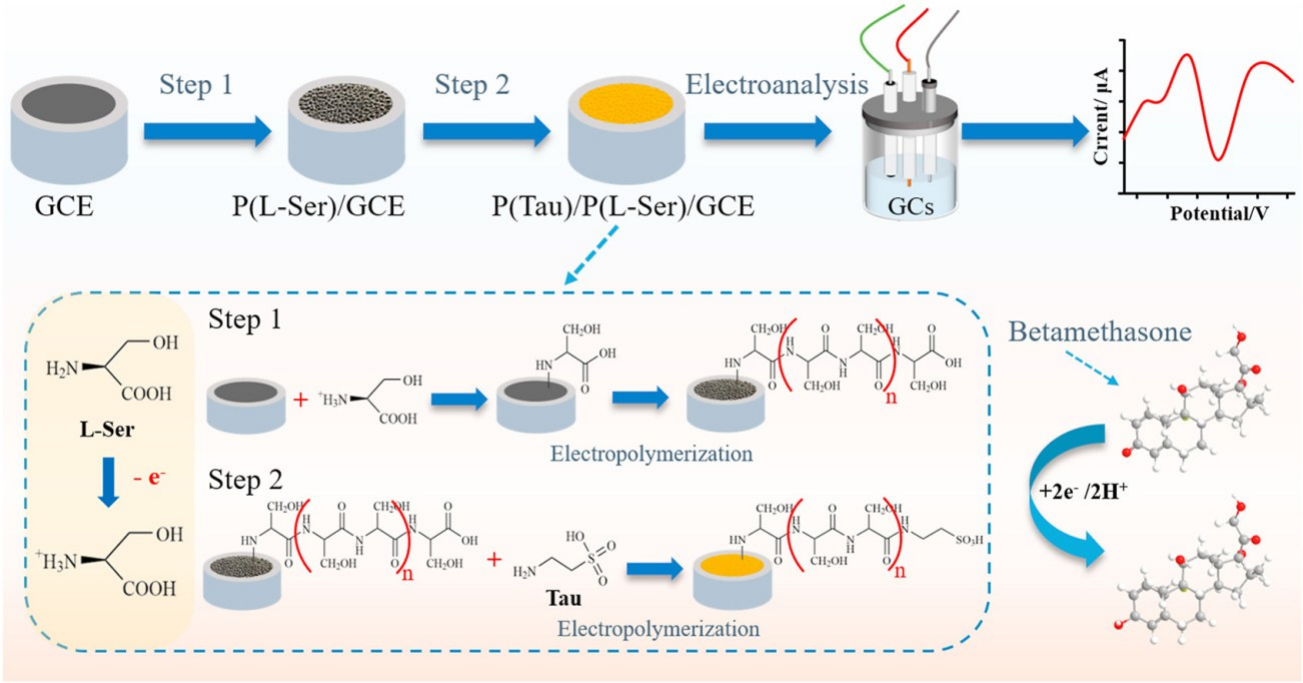
聚-L-丝氨酸/聚-牛磺酸/玻碳电极双层电聚合传感电极制备示意图^［94］^

近年来，传感检测、酶联免疫吸附法等快检技术在食品、生物等复杂样品中得到了广泛应用，它们在化妆品快筛中的研究和应用也逐步增多。Zhang等^［95］^基于糖皮质激素的环己-2，5-二烯-1-酮结构特征，建立了化妆品中地塞米松的快速免疫分析方法。该方法利用包被抗原与地塞米松抗体间的竞争性免疫吸附反应，通过掺杂镧系元素的上转换纳米粒子对抗体进行标记，借助目测观察或光密度扫描仪实现检测信号读取，对化妆品中地塞米松的检出限达到2.0 μg/kg。该研究通过特异性抗体识别与纳米材料信号放大技术的结合，实现了复杂基质中目标物的高灵敏检测；他们还以新西兰兔对氢化可的松免疫产生的非克隆抗体为基础，将黄体酮羧酸作为其竞争性合体，开发了一种同源竞争性间接酶联免疫吸附法^［96］^，用于测定化妆品中的氢化可的松，检出限为0.04 ng/mL。此外，Zhang等^［97］^使用基于荧光阅读器的条形检测试剂盒，以二氧化硅包覆掺杂有镧系元素的高发光上转换纳米粒子为探针、地塞米松衍生物作为包被抗原，通过结合位点保护策略，提高了上转换纳米粒子免疫层析法的灵敏度，实现了对曲安奈德的现场检测，包括前处理在内仅需10 min即可完成。Tao等^［98］^使用具有核壳结构的CdSe/CdS量子点作为荧光探针，利用氢化可的松抗原-CdSe/CdS量子点偶联物与游离氢化可的松同Fe_3_O_4_/Au纳米颗粒上固定的氢化可的松抗体之间的竞争性结合，可实现对化妆品中的氢化可的松特异性检测，LOD可达0.5 pg/mL。

化妆品中糖皮质激素的快速检测方法凭借其高效便捷的特点，在应对庞大样品量与现场筛查需求中展现出显著优势。然而，快检方法普遍存在灵敏度低于色谱-质谱联用技术、抗基质干扰能力弱及依赖特定试剂等问题。相较于实验室方法的高分离度与色谱-质谱联用的精准定性能力，快检技术更适用于初筛与现场应急，而复杂样品仍需结合色谱/质谱确证。未来化妆品快检方法的发展方向可以是开发微型化质谱设备以融合快检速度与质谱定性优势，优化纳米材料/生物探针提升免疫法的特异性与灵敏度，并借助人工智能算法实现多技术联用数据的自动解析，形成“现场快检初筛-实验室精密复核”的全链条监管体系，为化妆品安全提供高效、灵活且可靠的解决方案。

## 3 总结与展望

近年来，化妆品中糖皮质激素等禁限用组分滥用频繁，现阶段固相萃取等前处理技术与色谱、色谱-质谱等检测技术是化妆品质量监控的主要手段。糖皮质激素类物质结构多样，新的糖皮质激素不断被发现和使用，结合色谱、质谱、核磁等构建数据库，并结合大数据分析实现对非法添加糖皮质激素的快速筛查定性，对主动发现风险和监管预警具有重要意义。化妆品种类多样、基体复杂，这为微痕量糖皮质激素的分析检测提出了更高的要求，发展快速、高效和普适性的样品前处理技术，是化妆品中糖皮质激素高效分离分析的关键。同时，为应对日益壮大的化妆品市场监管要求，快速分析，尤其是适合现场快速筛查的分析技术，将成为化妆品中糖皮质激素检测的发展方向。高效样品前处理技术与紫外、荧光、表面增强拉曼光谱等高灵敏分析检测方法的一体化集成^［99-102］^，以及循环化学发光^［103，104］^等新的分析技术，已在食品、生物等领域得到成功应用，有望成为一类新的化妆品现场快速检测方法。
